# Intra- and Inter-week Variations of Well-Being Across a Season: A Cohort Study in Elite Youth Male Soccer Players

**DOI:** 10.3389/fpsyg.2021.671072

**Published:** 2021-04-09

**Authors:** Hadi Nobari, Maryam Fani, Filipe Manuel Clemente, Jorge Carlos-Vivas, Jorge Pérez-Gómez, Luca Paolo Ardigò

**Affiliations:** ^1^Department of Physical Education and Sports, University of Granada, Granada, Spain; ^2^HEME Research Group, Faculty of Sport Sciences, University of Extremadura, Cáceres, Spain; ^3^Sepahan Football Club, Isfahan, Iran; ^4^Department of Biological Sciences in Sport and Health, Faculty of Sports Sciences and Health, Shahid Beheshti University, Tehran, Iran; ^5^Escola Superior Desporto e Lazer, Instituto Politécnico de Viana Do Castelo, Rua Escola Industrial Comercial de Nun'Álvares, Viana Do Castelo, Portugal; ^6^Department of Neurosciences, Biomedicine and Movement Sciences, School of Exercise and Sport Science, University of Verona, Verona, Italy

**Keywords:** hooper index, playing position, monitoring, professional, performance, recovery

## Abstract

This study describes the weekly variations of well-being ratings relative to fatigue (wFatigue), stress (wStress), delayed-onset muscle soreness (wDOMS), sleep quality (wSleep), and Hooper questionnaire (wHQ) throughout the season. In addition, the well-being variables for the playing position in different moments of the season were discussed. Twenty-one elite young soccer players U17 took part in this study. From the beginning of the pre-season, well-being status was monitored daily by the HQ method throughout 36 weeks, including four periods: (1) pre-season, (2) early-season, (3) mid-season, and (4) end-season. Players trained at least 3 times per week throughout the season. The main outcome was that, in weeks 33 and 28, the highest [wFatigue: 15.85 ± 3.38 arbitrary units (AU); wHQ: 48.86 ± 9.23 AU] and the lowest (wFatigue: 5.38 ± 1.88 AU; wHQ: 20.43 ± 5.49 AU) wFatigue and wHQ occurred, respectively, although the lowest level of wDOMS happened in week 28 (4.86 ± 2.15 AU), while the highest wDOMS was observed in week 5 (14.65 ± 4.16 AU). The highest wSleep (13.00 ± 2.12 AU) and wStress (11.65 ± 2.92 AU) were observed in weeks 8 and 34, respectively, while the lowest wSleep (5.81 ± 2.29 AU) and wStress (3.76 ± 0.94 AU) were marked in week 29 coincidentally. In the HQ between every weekday, except recovery day, and the day of the match (MD), considerable highest HQ was only revealed in 2 days after MD in contrast to overall team comparison. In the present study, we observed that the well-being changes between different phases of the season as well as between weeks and days of the week with the MD are significant. These results provide a great point of view for coaches and practitioners about well-being variations over a season in elite youth soccer level. As a result, coaches will be more aware about non-functional overreaching and taking measures to prevent it.

## Introduction

Monitoring internal training load (TL) has been used extensively and well-discussed in sports, particularly in team sports (Clemente et al., 2017; Nobari et al., 2020b). Quantifying training is a common practice conducted in professional sports teams (Clemente et al., 2019a). Knowledge about the exact impacts of the TLs on youth athletes allows for the management and direction of the variation of stimulus, the optimization of the training individualization, a reduction in risk of injury, the early detection of bad overreaching, and the minimization of the possibility of overtraining syndrome (Wrigley et al., 2012; Gabbett et al., 2017). In addition, youth soccer players may experience different physical and physiological pressures related to their age-specific conditions that can cause premature injury or illness, that is why it is important to assess their monitoring training in these ages (Brink et al., 2010). The main purposes of monitoring are to determine the external and internal load imposed on youth athletes through training and to determine the acute and long-term implications of training (Bourdon et al., 2017; Clemente et al., 2019a, 2021). Mostly, TLs can be identified as either internal or external (Arslan et al., 2017). Internal load defines the physiological influence of training (e.g., effects of heart rate, blood lactate concentrations, or rating perceived exertion). External load usually describes the physical effects of training on players (e.g., distances covered at different speed thresholds, accelerations, decelerations, or jumps) (Rebelo et al., 2012; Wrigley et al., 2012). Furthermore, compared to internal physiological measures, such as heart rate and rating of perceived exertion, other measures of physiological status are less known. Recent literature has introduced the use of the Hooper questionnaire (HQ) (Charlot et al., 2016). HQ is a method based on self-analysis questionnaires involving the well-being ratings relative to fatigue, stress level, delayed-onset muscle soreness (DOMS), and sleep quality/disorders (Hooper and Mackinnon, 1995).

Indeed when athletes do not sufficiently respect the balance between training and recovery, non-functional overreaching (NFOR) can occur (Meeusen et al., 2013). The distinction between NFOR and overtraining syndrome will depend on the clinical outcome and exclusion diagnosis and is very difficult (Meeusen et al., 2013), but semantically, overreaching is an accumulation of training and/or no training stress, resulting in short-term decrement in performance capacity, with or without related physiological and psychological signs and symptoms of maladaptation, in which restoration of performance capacity may take from several days to several weeks (Meeusen et al., 2013; Nobari et al., 2021a). On the other hand, if coaches are aware of a series of signs of overtraining, they can recognize it, although it is accurate diagnosis during competition that causes a decline in performance. Some of these symptoms include decreased appetite, weight loss, headache and allergic responses, sleep disturbance, increased resting heart rate, premature injury, and fatigue (Meeusen et al., 2013).

Awareness of well-being is considered a useful sign for identifying NFOR (Noon et al., 2015). Studies in youth soccer players have shown a connection between declining perceptions of well-being and NFOR (Brink et al., 2012; Noon et al., 2015). Moreover, elite coaches try to prepare athletes with the suitable load that prevents acute or NFOR through the different moments of the season (Jones et al., 2017). Therefore, the HQ measured daily training session, not only allowing better detection of individual signs of pre-fatigue when interpreted along with the players' TLs [4] but also eventually adapting the scheduled TLs of the day in light of the players' status (i.e., amateur or professional players). This will eventually allow the staff and fitness coach to exactly schedule and adapt the TLs to reach optimal performance, with fit players, and to observe optimal weekly load distribution to ensure sufficient post-match recovery and prevent pre-match fatigue (Haddad et al., 2013; Nobari et al., 2020b).

Despite the fact that the above-mentioned research has enhanced our understanding of the variation of fatigue in different periods, namely, identifying some decreases in performance variables (Carling et al., 2015) and the association of TLs with quality-of-life variables, we believe that it is still necessary to cross in a single study the variables of TLs, perception of fatigue, stress, muscle soreness, and sleep and analyze such variance in different types of week (Clemente et al., 2017). Moreover, as we have said before, daily monitoring of internal load and wellness status can help coaches to know more about the impact of training on their players and may help them to prevent risk of NFOR and injury. The data analysis should combine the analysis of daily and weekly data for the different players and the team. Each analysis provides different perspectives about the effective stimulus that is perceived by each athlete. These protocols provide helpful information to handle injury prevention programs (Clemente et al., 2019c; Nobari et al., 2020b). Therefore, this study has three aims: (1) to describe the weekly patterns (within-week comparisons) of well-being across the season with the HQ in terms of weekly fatigue, weekly stress, weekly sleep, and weekly DOMS in elite youth soccer players, (2) to analyze the differences of well-being variables between early-, mid-, and end-season periods, and (3) to compare the well-being variables for playing positions in different moments of the season.

## Methods and Material

### Participants

Twenty-one elite young soccer players participated in this study (mean ± standard deviation; age, 16.1 ± 0.2 years; height, 176.8 ± 5.6 cm; body mass, 67.3 ± 5.7 kg; BMI, 21.5 ± 1.4 kg/m^2^; VO_2max_, 47.6 ± 3.8 ml kg^−1^ min^−1^). The participants were the main players of the professional team under-17 (U17). To analyze the differences between playing positions, we differentiate between five fullback (FB), four center half (CH), four center midfielder (CM), five winger (WG), and three forward (FW) (Nobari et al., 2021b). The inclusion criteria in this study were as follows: (i) players who participated in at least 90% of training seasons, (ii) players were not allowed to participate in another training plan along with this study, (iii) each player who was not participating in the match during a week was practicing in a separate session, without the ball or small side games, and (iv) goalkeepers were not included in the statistical analyses. The study was conducted in accordance with the Declaration of Helsinki. Prior to starting, the players and their parents signed an informed consent to participate in this study, which was approved by the Ethics Committee of the Sport Sciences Research Institute (IR.SSRC.REC.1399.060).

### Experimental Approach to the Problem

This study is a descriptive–longitudinal study that monitored a full-season for a soccer team. Daily monitoring was observed by players for 36 weeks from the beginning of the preparation season. The full season was divided into four periods according to the team competition schedule: (1) pre-season, weeks (W) 1 to W5, (2) early-season, W6 to W13, (3) mid-season, W14 to W31, and (4) end-season, W32 to W36 (Table 1). To analyze the differences between the three in-season periods and by playing position in every in-season periods, all well-being variables were considered for analysis. The description of the typical microcycle pattern and its corresponding analyses were conducted considering only the data from those competition weeks with the most repeated training pattern and that included only one match. The players trained at least 3 times per week during the season. The players had been using the scale of HQ for the last 3 years. Daily sleep, stress, fatigue, and DOMS status data were collected to report changes in weekly wellness status (i.e., HQ) (Hooper and Mackinnon, 1995). The Intermittent Fitness Test (IFT) 30-15 was used to report the participants' level of readiness to calculate their maximum oxygen uptake (VO_2max_).

**Table 1:** Monitoring during the full season.

**Year**	**2019**										**2020**
**Months**	**May**	**June**	**July**	**Aug**	**Aug**	**Sept**	**Oct**	**Nov**	**Dec**	**Dec**	**Jan**
Weeks	1–4	5–8	9–12	13	14–16	17–20	21–24	25–28	29–31	32	33–36
TS	20	23	19	4	15	21	20	18	14	5	20
**Phase**	**First PP**	**Regional league**								**Second PP**	**Best of Iran (National)**
**Periods**	**Pre-season**	**Early-season**			**Mid-season**					**End-season**	
OG	–	–	–	–	3	3	4	5	3	–	8
NOG	2	3	3	–	–	–	–	–	–	–	–

TS, training session; PP, preparation phase; OG, official games number; non-official games number; W, week.

### Anthropometric Measurements

Anthropometric variables such as standing height (Seca model 213, Germany, with an accuracy of ±5 mm) and weight (Seca model 813, the UK with an accuracy of 0.1 per kilogram) were measured by the techniques provided by the International Society for the Advancement of Kinanthropometry (Norton and Olds, 1996; Rahmat Ali Jafari et al., 2016). These measurements were done between 8 and 11 A.M. (Arazi et al., 2015).

### Performance Test

The IFT 30-15 was used to calculate the VO_2max_ of the players. The test consists of 30-s shuttle runs interspersed with 15-s passive recovery periods on a 40-m straight runway. The running velocity starts at 8 km/h^−1^ and is increased by 0.5 km/h^−1^ at every 45-s stage thereafter (Buchheit, 2010). Three lines need to be setup for the 30-15 test. Line A should be 20 m away from line B, and line C should be 20 m away from line B and therefore 40 m from line A. For warm-up in these tests, the players performed 10 min of standard warm-up, such as jogging, dynamic stretching, some ABC run drills, and submaximal short speeds under the supervision of fitness coach of the team. After the warm-up, all the players were divided into groups of four. Standing on line A, after hearing a “Ready, go!” signal from the speakers, they started running to line B and C for 30 s. After that, they would take themselves to another line for the next step. If the participants were totally exhausted or if they could not achieve the 2-m lines for three consecutive times, they can stop on their own volition. This stage level was recorded as velocity of IFT (VIFT). This test was performed in the pre-season and then calculated by the relevant formula: VO_2max_ (ml kg^−1^ min^−1^) = 28.3–(2.15 × 1)–(0.741 × 17 yrs.)–(0.0357 × weight) + (0.0586 × 17 years × VIFT) + (1.03 × VIFT). VIFT was considered as the final speed of the player in the exhaustion test (Buchheit, 2010).

### Well-Being Status Monitoring

The HQ is a self-report questionnaire based on a seven-point scale involving the well-being status relative to stress, fatigue, DOMS, and sleep quality (Clemente et al., 2017; Nobari et al., 2020a). The HQ is the summation of four subjective ratings (Hooper and Mackinnon, 1995). HQ was applied 30 min before each session. In this questionnaire, number one means good condition, and number seven means very bad condition. Prior to the research, the players were familiarized with the scale (at least 3 years of using HQ). The following accumulated data were obtained for each variable by the sum of a week: (i) wStress, (ii) wFatigue, (iii) wDOMS, (iv) wSleep, and (v) wHQ. The data collection occurred individually to avoid the players from hearing the scores of other teammates. The daily data register was made in Excel.

### Statistical Analysis

Descriptive statistics are presented as mean and standard deviation (SD). Shapiro–Wilk and Levene's tests were executed for verifying data normality and homogeneity, respectively. Changes between the three in-season periods were assessed using a repeated-measures analysis of variance (ANOVA), followed by Bonferroni *post-hoc* test for pairwise comparisons. Partial eta-square ($\eta_{\text{p}}^{2}$) was calculated as the effect size of the repeated-measures ANOVA. Similar procedures were applied for analyzing the possible differences between every weekday and the match day (MD) in HQ during a common competition microcycle. Additionally, a one-way ANOVA was applied to compare the different well-being variables, by playing position, in each in-season period. Hedges' *g* effect size with 95% confidence interval was also calculated to determine the magnitude of pairwise comparisons for between-period comparison. The Hopkins' thresholds for effect size statistics were used as follows: ≤ 0.2, trivial; >0.2, small; >0.6, moderate; >1.2, large; >2.0, very large; and >4.0, nearly perfect 4. The significance level was set at *P* ≤ 0.05. The Statistical Package for Social Sciences (SPSS, version 25.0; IBM SPSS Inc., Chicago, IL) was used for computations.

## Results

**Figure 1:**
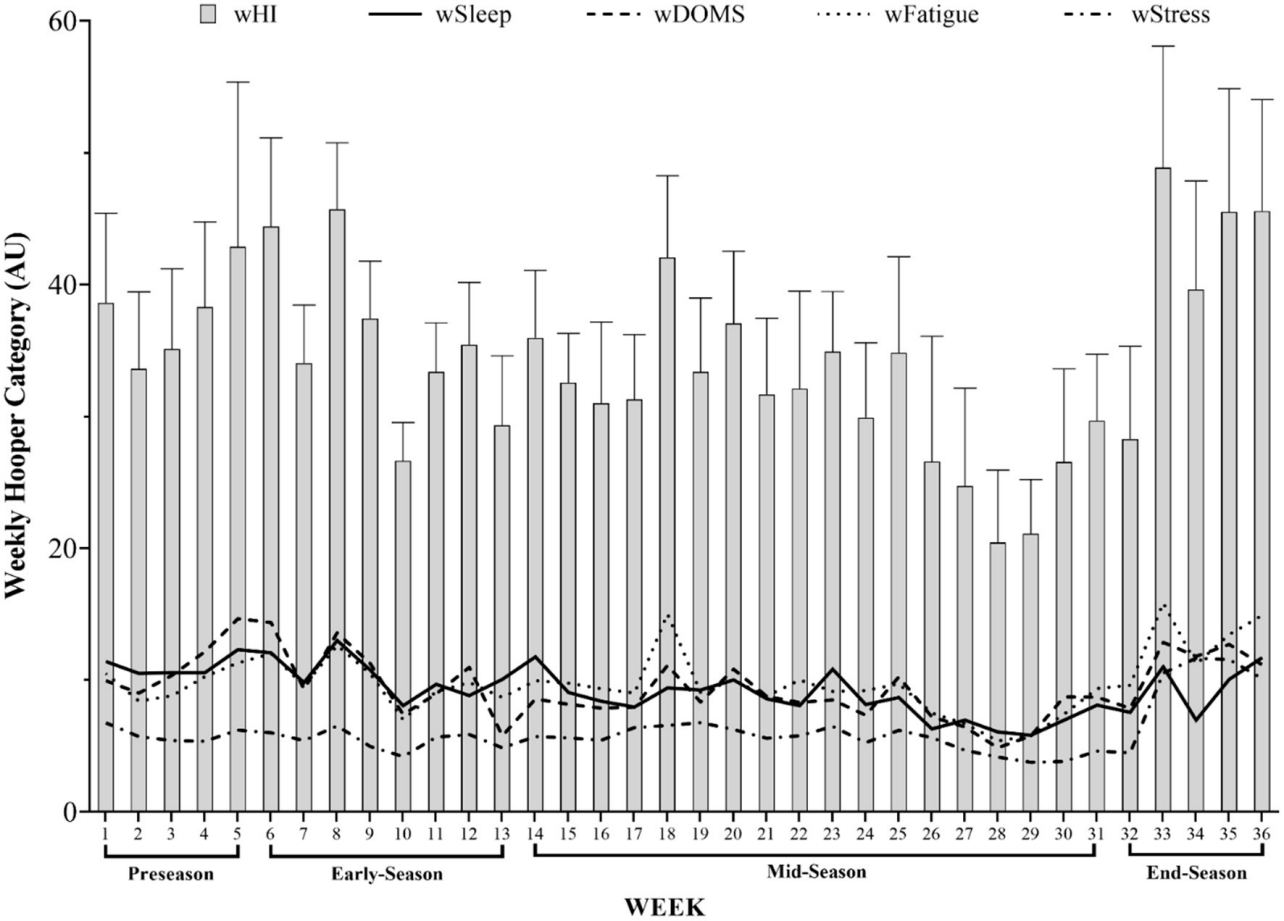
Description of weekly patterns for well-being variables across the season. wSleep, weekly sleep; wDOMS, weekly muscle soreness; wFatigue, weekly fatigue; wStress, weekly stress; wHQ, weekly Hooper questionnaire.

Figure 1 shows the weekly patterns for well-being variables (wSleep, wDOMS, wFatigue, wStress, and wHQ) across the full season and its periods. Coincidentally, the highest and the lowest wFatigue [↑: 15.85 ± 3.38 arbitrary units (AU); ↓: 5.38 ± 1.88 AU] and wHQ (↑: 48.86 ± 9.23 AU; ↓:20.43 ± 5.49 AU) occurred in weeks 33 and 28, respectively. The lowest wDOMS also happened in week 28 (4.86 ± 2.15 AU); however, the highest wDOMS was observed in week 5 (14.65 ± 4.16 AU). Besides this, the highest wSleep (13.00 ± 2.12 AU) and wStress (11.65 ± 2.92 AU) were presented in week 8 and week 34, respectively, while the lowest wSleep (5.81 ± 2.29 AU) and wStress (3.76 ± 0.94 AU) were coincidentally observed in week 29.

**Figure 2:**
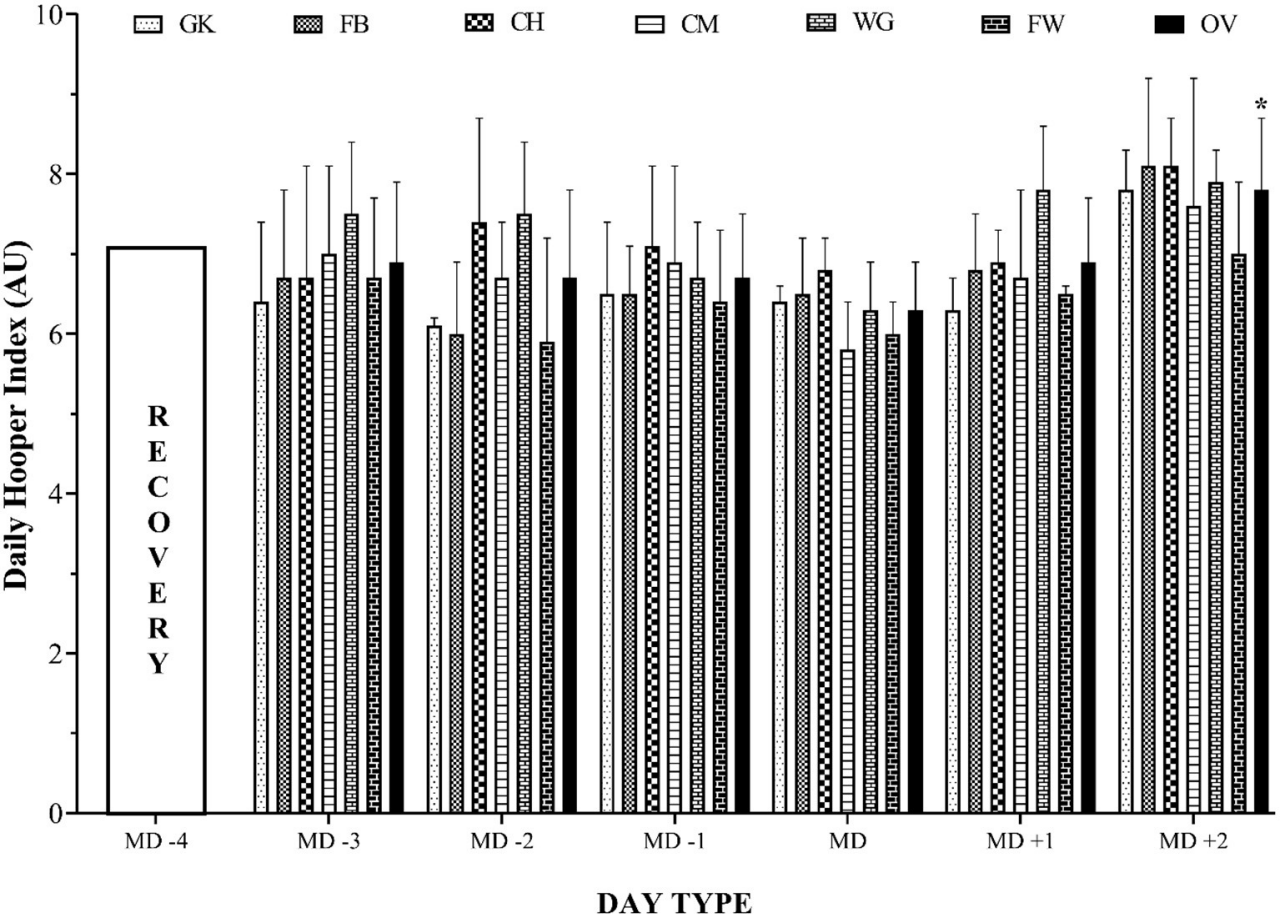
Daily pattern and comparisons between every weekday and the match day in the Hooper questionnaire during a common competition microcycle for the overall team and by playing position. GK, goalkeeper; FB, fullback; CH, center half; CM, center midfielder; WG, winger; FW, forward. *Significant differences for *P* ≤ 0.05 compared to MD, match day.

Figure 2 displays the daily pattern and comparisons between every weekday and the MD in the HQ during a common competition microcycle for the overall team and by playing position. Repeated-measures ANOVA only revealed significant highest HQ in MD^+2^ (2 days after match day) (*P* < 0.001) compared to MD for overall team comparison. No differences were found for the rest of the overall team comparisons and neither for analyses by playing position (*P* > 0.05).

Results of repeated-measures ANOVA revealed differences between season periods in wSleep (*P* < 0.001, $\eta_{\text{p}}^{2}$ = 0.378), wDOMS (*P* < 0.001, $\eta_{\text{p}}^{2}$ = 0.664), wFatigue (*P* < 0.001, $\eta_{\text{p}}^{2}$ = 0.743), wStress (*P* < 0.001, $\eta_{\text{p}}^{2}$ = 0.916), and wHQ (*P* < 0.001, $\eta_{\text{p}}^{2}$ = 0.873). Table 2 presents the pairwise comparisons between all in-season periods for wSleep, wDOMS, wFatigue, wStress, and wHQ. Overall, the end-season presented a significantly greater wDOMS, wFatigue, wStress, and wHQ compared to early-season (wDOMS: *P* = 0.026, *g* = 0.82; wFatigue: *P* < 0.001, *g* = 1.96; wStress: *P* < 0.001, *g* = 4.76; wHQ: *P* < 0.001, *g* = 2.79) and mid-season (wDOMS: *P* < 0.001, *g* = 2.35; wFatigue: *P* < 0.001, *g* = 2.68; wStress: *P* < 0.001, *g* = 4.67; wHQ: *P* < 0.001, *g* = 3.87). The early-season had a likewise significantly greater wDOMS (*P* < 0.001, *g* = −1.54), wFatigue (*P* = 0.003, *g* = −0.91), and wHQ (*P* < 0.001, *g* = −1.63) compared to mid-season. However, no differences in wStress (*P* = 1.000, *g* = 0.09) were reported when the early-season and mid-season periods were compared. Besides this, meaningful greater values of wSleep were observed for early-season compared with mid-season (*P* = 0.001, *g* = −1.16) and for mid-season compared with end-season (*P* = 0.002, *g* = 0.62). However, no differences in wSleep were observed when early-season and end-season were compared (*P* = 0.551, *g* = −0.35).

**Table 2:** Comparison between season periods, considering well-being variables.

	**Season period**	**Comparison**	**%Difference (95% CI)**	***P***	**Hedges' *g* (95% CI)**
wSleep (AU)	EarS: 10.16 (1.60)	EarS *vs*. MidS	−19.2 (−27.1 to −10.4)	**0.001**	−1.16 (−1.73 to −0.38)
	MidS: 8.29 (1.86)	EarS *vs*. EndS	−7.1 (−16.0 to 2.9)	0.551	−0.35 (−0.99 to 0.29)
	EndS: 9.51 (2.00)	MidS *vs*. EndS	15.0 (7.3 to 23.2)	**0.002**	0.62 (−0.03 to 1.27)
wDOMS (AU)	EarS: 10.02 (1.29)	EarS *vs*. MidS	−19.8 (−27.2 to −11.7)	** <0.001**	−1.54 (−2.26 to −0.81)
	MidS: 8.05 (1.22)	EarS *vs*. EndS	11.0 (2.9 to 19.7)	**0.026**	0.82 (0.16 to 1.48)
	EndS: 11.12 (1.34)	MidS *vs*. EndS	38.5 (29.0 to 48.6)	** <0.001**	2.35 (1.52 to 3.17)
wFatigue (AU)	EarS: 9.92 (1.23)	EarS *vs*. MidS	−11.9 (−17.7 to −5.6)	**0.003**	−0.91 (−1.58 to −0.24)
	MidS: 8.76 (1.26)	EarS *vs*. EndS	30.8 (18.6 to 44.2)	** <0.001**	1.96 (1.19 to 2.74)
	EndS: 13.00 (1.79)	MidS *vs*. EndS	48.4 (36.7 to 61.0)	** <0.001**	2.68 (1.81 to 3.56)
wStress (AU)	EarS: 5.44 (0.31)	EarS *vs*. MidS	−0.5 (−2.9 to 4.0)	**1.000**	0.09 (−0.55 to 0.72)
	MidS: 5.47 (0.36)	EarS *vs*. EndS	77.7 (66.2 to 89.9)	** <0.001**	4.76 (3.51 to 6.00)
	EndS: 9.73 (1.21)	MidS *vs*. EndS	76.8 (64.6 to 89.9)	** <0.001**	4.67 (3.44 to 5.90)
wHQ (AU)	EarS: 35.55 (2.44)	EarS *vs*. MidS	−14.3 (−19.1 to −9.2)	** <0.001**	−1.63 (−2.37 to −0.90)
	MidS: 30.58 (3.43)	EarS *vs*. EndS	21.9 (16.6 to 27.5)	** <0.001**	2.79 (1.89 to 3.68)
	EndS: 43.35 (3.01)	MidS *vs*. EndS	42.2 (35.5 to 49.3)	** <0.001**	3.87 (2.80 to 4.95)

AU, arbitrary units; wSleep, weekly sleep in AU; wDOMS, weekly muscle soreness in AU; wFatigue, weekly fatigue in AU; wStress, weekly stress in AU; wHQ, weekly Hooper questionnaire in AU; Ear-S, early-season period; Mid-S, mid-season period; End-S, end-season period; P, p-value at alpha level 0.05; Hedges' g (95% CI), Hedges' g effect size magnitude with 95% confidence interval.

*Significant differences (p ≤ 0.05) are highlighted in bold*.

The comparisons between the different playing positions are displayed in Tables 3–5 for the early-season, mid-season, and end-season, respectively. Overall, the results of one-way ANOVA revealed no significant differences between playing positions for any well-being variable in the different in-season periods (*P* > 0.050). Thus, *post-hoc* tests were not applied for analyzing pairwise comparisons.

**Table 3:** Outcomes of ANOVA for well-being variables during early-season, considering playing positions.

	**Playing position**	***F***	***P***
wSleep (AU)	FB: 9.93 (1.79)	0.184	0.943
	CH: 10.25 (0.74)		
	CM: 9.97 (2.51)		
	WG: 10.45 (1.81)		
	FW: 10.88 (0.88)		
wDOMS (AU)	FB: 10.53 (1.90)	0.569	0.689
	CH: 9.80 (0.57)		
	CM: 9.72 (1.63)		
	WG: 10.73 (1.09)		
	FW: 9.67 (1.00)		
wFatigue (AU)	FB: 10.75 (1.67)	1.808	0.177
	CH: 9.54 (0.62)		
	CM: 10.41 (0.67)		
	WG: 9.20 (1.11)		
	FW: 9.33 (0.44)		
wStress (AU)	FB: 5.28 (0.16)	1.663	0.208
	CH: 5.45 (0.38)		
	CM: 5.28 (0.16)		
	WG: 5.68 (0.34)		
	FW: 5.46 (0.29)		
wHQ (AU)	FB: 36.48 (3.48)	0.215	0.926
	CH: 35.04 (1.20)		
	CM: 35.38 (3.81)		
	WG: 36.05 (2.10)		
	FW: 35.33 (0.47)		

*AU, arbitrary units; AU, arbitrary units; wSleep, weekly sleep in AU; wDOMS, weekly muscle soreness in AU; wFatigue, weekly fatigue in AU; wStress, weekly stress in AU; wHQ, weekly Hooper questionnaire in AU; FB, fullback; CH, center half; CM, center midfielder; WG, winger; FW, forward; P, p-value at alpha level 0.05*.

**Table 4:** Outcomes of ANOVA for well-being variables during mid-season, considering playing positions.

	**Playing position**	***F***	***P***
wSleep (AU)	FB: 7.70 (1.58)	1.173	0.360
	CH: 8.70 (1.65)		
	CM: 7.53 (1.50)		
	WG: 9.62 (1.92)		
	FW: 7.74 (2.20)		
wDOMS (AU)	FB: 7.56 (1.00)	0.901	0.486
	CH: 8.29 (0.97)		
	CM: 8.10 (1.22)		
	WG: 8.99 (1.62)		
	FW: 7.89 (1.19)		
wFatigue (AU)	FB: 8.77 (1.39)	2.569	0.078
	CH: 8.67 (1.28)		
	CM: 10.07 (0.46)		
	WG: 9.28 (1.30)		
	FW: 7.39 (0.63)		
wStress (AU)	FB: 5.31 (0.08)	1.374	0.287
	CH: 5.80 (0.50)		
	CM: 5.40 (0.34)		
	WG: 5.43 (0.29)		
	FW: 5.48 (0.34)		
wHQ (AU)	FB: 29.33 (3.52)	1.199	0.349
	CH: 31.46 (3.56)		
	CM: 31.10 (2.88)		
	WG: 32.93 (2.85)		
	FW: 28.50 (3.66)		

*AU, arbitrary units; AU, arbitrary units; wSleep, weekly sleep in AU; wDOMS, weekly muscle soreness in AU; wFatigue, weekly fatigue in AU; wStress, weekly stress in AU; wHQ, weekly Hooper questionnaire in AU; FB, fullback; CH, center half; CM, center midfielder; WG, winger; FW, forward; P, p-value at alpha level 0.05*.

**Table 5:** Outcomes of ANOVA for well-being variables during end-season, considering playing positions.

	**Playing position**	***F***	***P***
wSleep (AU)	FB: 9.80 (2.46)	0.240	0.911
	CH: 9.85 (2.18)		
	CM: 9.00 (1.14)		
	WG: 8.44 (4.59)		
	FW: 8.47 (2.01)		
wDOMS (AU)	FB: 11.04 (1.37)	0.586	0.677
	CH: 10.50 (0.82)		
	CM: 11.20 (1.07)		
	WG: 11.90 (2.53)		
	FW: 11.93 (1.29)		
wFatigue (AU)	FB: 13.00 (1.48)	0.758	0.568
	CH: 13.80 (1.99)		
	CM: 13.70 (0.26)		
	WG: 12.15 (2.85)		
	FW: 12.13 (1.14)		
wStress (AU)	FB: 9.52 (1.68)	0.448	0.773
	CH: 9.80 (1.25)		
	CM: 9.75 (1.41)		
	WG: 8.16 (4.06)		
	FW: 10.07 (1.30)		
wHQ (AU)	FB: 43.36 (2.31)	1.197	0.350
	CH: 43.95 (3.34)		
	CM: 43.65 (2.73)		
	WG: 35.56 (13.44)		
	FW: 42.60 (0.92)		

*AU, arbitrary units; AU, arbitrary units; wSleep, weekly sleep in AU; wDOMS, weekly muscle soreness in AU; wFatigue, weekly fatigue in AU; wStress, weekly stress in AU; wHQ, weekly Hooper questionnaire in AU; FB, fullback; CH, center half; CM, center midfielder; WG, winger; FW, forward; P, p-value at alpha level 0.05*.

## Discussion

In this study, daily monitoring was noted by players from the beginning of the preparation season for 36 weeks. The full season was divided into four periods. The three aims of this study were to (i) describe the weekly patterns (within-week comparisons) of well-being across the season, (ii) analyze the differences of well-being variables between early-, mid-, and end-season periods, and (iii) compare well-being variables for playing positions in different moments of the season. However, this is the first study that investigated the variance of wellness during the season in different player positions indicating elite youth soccer players.

The main result was that, in week 33 and week 28, the highest and the lowest wFatigue and wHQ occurred, respectively. Although the low level of wDOMS happened in week 28, the highest wDOMS was observed in week 5. The highest wSleep and wStress were demonstrated in week 8 and week 34, correspondingly, while the lowest wSleep and wStress were coincidentally observed in week 29. The daily pattern and comparisons between every weekday and the MD in the HQ also only revealed significant highest HQ in MD^+2^ compared to MD for overall team comparison.

This study is about well-being variables in weekly patterns and different periods of a season. The result indicated that the highest wStress, wFatigue, and wHQ occurred in end-season, and the lowest of them was observed in mid-season. It can be described that, at the end-season, due to the high sensitivity of the final competitions, the pressure and intensity of the competitions and the effort to win the championship could influence the results. Similar attitudes are found in some studies, where RPE and salivary cortisol during were enhanced during the final championship matches compared with during regular competition in elite young volleyball players (Moreira et al., 2013). Cumulative fatigue from TLs during the season in youth rugby players may also affect the ratings for wStress, wHQ, and wFatigue, causing them to rise at the end of the season (Oliver et al., 2015).

In addition, in the mid-season, no significant changes were observed for well-being indicators. In most indicators (wFatigue, wSleep, wHQ, wDOMS, and wStress), it was even lower than at any other time during the season. As stated in a study, the daily perceived ratings of sleep quality and muscle soreness have been found to be statistically significantly correlated with daily training load during the pre-season training period in elite Australian Football League (AFL) players (Buchheit et al., 2013; Moalla et al., 2016). In contrast, the relationship between daily training load and perceived ratings of sleep quality and muscle soreness was obvious and not significant in the other study. This may partly reflect the fact that previous observations in AFL players were made during the pre-season period, when the high volume and intensity of training may lead to greater disturbances in perceived ratings of sleep and soreness. In soccer, the high frequency of competition during the in-season phase confirmed that training is more focused on recovery and maintaining physical fitness, which may lead to lesser changes in perceived ratings of sleep and soreness across a typical training week (Thorpe et al., 2015). As we have seen in the present study, the highest number of muscle soreness was in the early-season, as a result of the high training volume, and fortunately, the muscle soreness continued to decrease due to the players' adaptation to training.

The results that related to sleep and stress in the present study were close to each other (Fernández-Fernández et al., 2015) so that their significant changes are related to the early- and end-season. Based on the findings, it seems that the importance and intensity of competition in the end-season have a significant impact on the quality of sleep and increase player stress (Nédélec et al., 2012, 2015) compared with the effects of different intensities (moderate: 60% and high: 80% heart rate reserve) of 40 min of pre-sleep treadmill running (9:20–10:00 p.m.) on sleep onset with a control condition. Compared with the non-exercise control condition, the sleep-onset latency was significantly longer in the high-intensity exercise condition. In addition, the total sleep time was significantly shorter, while sleep efficiency was significant lower following a high-intensity exercise compared with non-exercise. A significant difference was also observed in the subjective scores of “ease of going to sleep” between high-intensity exercise and non-exercise condition (Nédélec et al., 2015). Therefore, due to the high intensity of the final games of the end-season as well as maintaining the position and trying to be the main player at the early-season, it may have affected the quality of sleep and subsequently the stress of the players. On the other hand, a similar result that we encountered in the current study is that sleep and stress fluctuations in mid-seasons were less than in the early- and end-season, as in a study on elite volleyball players the amount of stress level in mid-season was less, which showed that the professional level of the players caused them to have better control over their mental state (Clemente et al., 2019c).

For the daily pattern and comparisons between every weekday and the MD in the HQ during a common competition macrocycle for the overall team and by playing position, no differences were found for the rest of the overall team comparisons, neither for analyses by playing position, and we only saw a significant highest HQ in MD^+2^ compared to MD for overall team comparison. These findings can be related to post-match DOMS, in fact presenting a DOMS not only in the immediate MD^+1^ but also as long as MD^+2^ after the match caused by the inflammation levels (Clemente et al., 2019b). This result is valuable because it shows that recovery on the day of rest is more important than any other factor in the whole macrocycle, and neglecting it increases cumulative fatigue and ultimately decreases performance and causes injury.

Moreover, we tried to examine the changes in the wellness status of the players between the days of the week and between the weeks during the season due to the fact that weekly data prepare information about the accumulative impact of microcycle. Elite athletes required an accumulative training load that causes them to attain the proper stimulus (Nobari et al., 2020b,c). The accumulative training load of elite athletes in a long season involves the fact that the athletes' functional over-reaching is a noticeable section of their training. The analysis of the weekly data demonstrates better the effect of training on the athletes. However, these results show that coaches should use mainly weekly values (e.g., variation from week to week) as an approach to analyze the effect of training on athletes; it must be considered that once the risk of overreaching is detected in the team, athletes' daily information will be necessary to monitor them and have them abstain from reaching NFOR (Clemente et al., 2019c).

Regarding comparison on well-being variables for playing positions in different moments of the season, in different studies, physiological differences in different positions in different players have been reported (Thelwell et al., 2006), but in the present study, there were no differences in well-being variables. It seems that this could be because these players are young at a certain age and their understanding about the well-being situation is the same. In addition, in the present study, only the competition season was considered. In the match season, the well-being variables are closer to each other because all players are under the same pressure at different moments such as stress, decreased quality of sleep, and so on. Ultimately, possibly examining the well-being variables in the preparation season can provide us with more information.

This study has limitations that need to be considered. First, we measured only one soccer team in the youth age category. It is better to compare several teams with different age groups. Second, it is better to collect this information on the day of recovery because it will provide more accurate information about the wellness of the players during the week. However, this is the first study to look at changes in the well-being of different players between days and weeks during the season. Therefore, more studies should be done in different teams and different countries to generalize the results.

## Conclusion

The well-being changes between different phases within the season as well as between weeks and days of the week with the MD are significant. In general, the amount of changes in player welfare indicators at the beginning of the season and at the end of the season is impressive, but these changes were less fluctuating in the middle of the season. Also, the well-being indicators did not differ much between different players, but the remarkable point was that the amount of wDOMS on the MD^+2^ was more than during other days compared to MD, which shows the importance of recovery up to 48 h after a match. The general purpose of designing and examining these hypotheses is to make the coaches more aware of the well-being of the players and to consider the appropriate load during the days, weeks, and finally the months of the season. Because regardless of the well-being conditions, the syndrome NFOR becomes apparent and eventually leads to overtraining, and this is the beginning of serious injuries and failure.

## Data Availability Statement

The raw data supporting the conclusions of this article will be made available by the authors, without undue reservation.

## Ethics Statement

The studies involving human participants were reviewed and approved by Ethics Committee of the Sport Sciences Research Institute. Written informed consent to participate in this study was provided by the participants' legal guardian/next of kin.

## Author Contributions

HN, FC, and JP-G designed the study and drafted the paper. HN and MF performed the experiments. HN, MF, LA, and JC-A participated in the data analysis and drafted the manuscript. HN, LA, JP-G, FC, and MF revised the critical manuscript. All the authors read and approved the final version of the manuscript.

## Conflict of Interest

The authors declare that the research was conducted in the absence of any commercial or financial relationships that could be construed as a potential conflict of interest. The reviewer EA declared a past co-authorship with one of the authors FC.
